# Supplementary material for: Canonical Wnt Pathway Is Involved in Chemoresistance and Cell Cycle Arrest Induction in Colon Cancer Cell Line Spheroids
Source: Int J Mol Sci. 2023 Mar 9;24(6):5252. doi: 10.3390/ijms24065252 (PMC10049556; doi:10.3390/ijms24065252)
Supplement: Supplementary file 1 [file ijms-24-05252-s001.zip › ijms-2158674-supplementary.pdf]

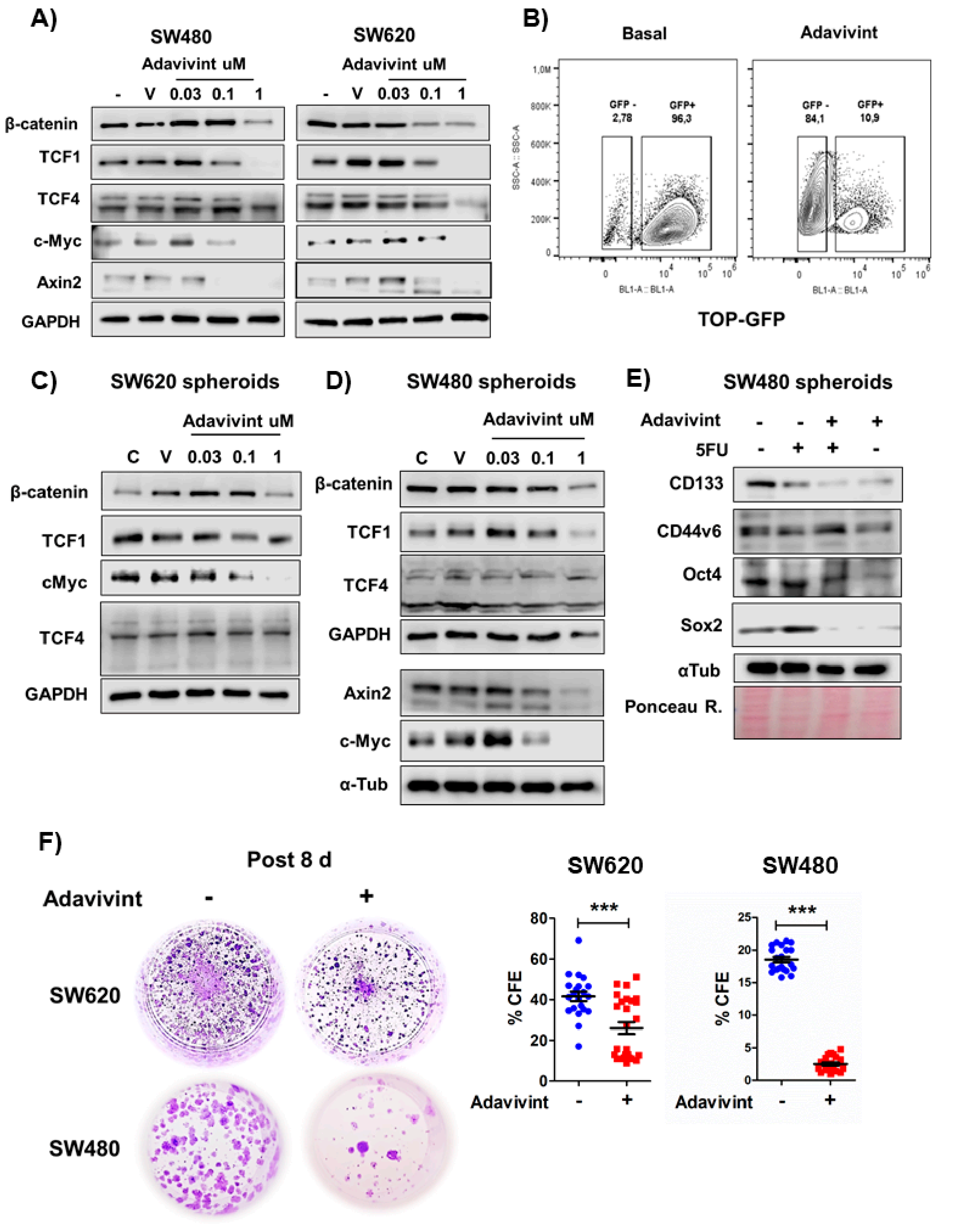

Figure S1. Adavivint inhibits the canonical Wnt pathway and clonogenicity of SW480 and SW620 cell lines. We determined the levels of β-catenin, TCF1, TCF4, c-Myc, and Axin2 through Western blot in monolayer (A) and spheroid cultures (C and D) of SW480 and SW620 cell lines treated with different doses of Adavivint for 48h or 72h, respectively. (B) β-catenin/TCF-mediated transcriptional activity was determined in SW480-spheroids from sorted cells that were transduced with construct TOP-GFP.

Spheroids were treated with 1μM Adavivint for 72h, and fluorescence was measured by flow cytometry in dissociated spheroids cells. (E) Immunoblots of cancer stem markers such as CD133, CD44v6, Sox2, and Oct4 of SW480-spheroids treated with or without Adavivint for 72h and subsequently with or without 5FU for 72h. (F) The clonogenic capacity of SW620 and SW480 cells derived from spheroids treated with Adavivint was evaluated on the 8th day post-treatment (free-drug) under dependent-anchorage conditions (% CFE, colony formation efficiency). Student's t-test, *** $p < 0.0001$.
